# Talking about Emotion: Prosody and Skin Conductance Indicate Emotion Regulation

**DOI:** 10.3389/fpsyg.2013.00260

**Published:** 2013-05-10

**Authors:** Moritz Matejka, Philipp Kazzer, Maria Seehausen, Malek Bajbouj, Gisela Klann-Delius, Winfried Menninghaus, Arthur M. Jacobs, Hauke R. Heekeren, Kristin Prehn

**Affiliations:** ^1^Cluster of Excellence “Languages of Emotion,” Freie UniversitätBerlin, Germany; ^2^Dahlem Institute for Neuroimaging of Emotion, Freie UniversitätBerlin, Germany; ^3^Department of Education and Psychology, Freie UniversitätBerlin, Germany; ^4^Department of Psychiatry, Campus Benjamin Franklin, CharitéBerlin, Germany; ^5^Department of Linguistics, Institut für Deutsche und Niederländische Philologie, Freie Universität BerlinBerlin, Germany; ^6^Max Planck Institute for Empirical AestheticsFrankfurt am Main, Germany

**Keywords:** emotion regulation, verbalization, IAPS, skin conductance, voice

## Abstract

Talking about emotion and putting feelings into words has been hypothesized to regulate emotion in psychotherapy as well as in everyday conversation. However, the exact dynamics of how different strategies of verbalization regulate emotion and how these strategies are reflected in characteristics of the voice has received little scientific attention. In the present study, we showed emotional pictures to 30 participants and asked them to verbally admit or deny an emotional experience or a neutral fact concerning the picture in a simulated conversation. We used a 2 × 2 factorial design manipulating the focus (on emotion or facts) as well as the congruency (admitting or denying) of the verbal expression. Analyses of skin conductance response (SCR) and voice during the verbalization conditions revealed a main effect of the factor focus. SCR and pitch of the voice were lower during emotion compared to fact verbalization, indicating lower autonomic arousal. In contradiction to these physiological parameters, participants reported that fact verbalization was more effective in down-regulating their emotion than emotion verbalization. These subjective ratings, however, were in line with voice parameters associated with emotional valence. That is, voice intensity showed that fact verbalization reduced negative valence more than emotion verbalization. In sum, the results of our study provide evidence that emotion verbalization as compared to fact verbalization is an effective emotion regulation strategy. Moreover, based on the results of our study we propose that different verbalization strategies influence valence and arousal aspects of emotion selectively.

## Introduction

Emotion regulation, that is the up- or down-regulation of positive or negative emotion, has primarily been investigated by focusing on *cognitive control mechanisms* (Lazarus and Folkman, 1984; Gross, 1998a, 2007). The model by Gross, for instance, distinguishes different control strategies by the time of occurrence: antecedent-focused strategies precede emotional responses, whereas response-focused strategies are employed to modulate an already initiated emotional response. Antecedent-focused strategies comprise situation selection, situation modification, attention deployment, and cognitive change. A form of cognitive change that has received particular attention in the research literature is the so-called “reappraisal.” Response-focused strategies include, among others, the suppression of emotional expressions. There is evidence that both reappraisal and suppression of emotional display result in changes of self-reported emotional experience and modify psycho-physiological responses (Jackson et al., 2000; Demaree et al., 2006). Recent studies show that these changes are accompanied by increased activity in the dorsal anterior cingulate cortex and prefrontal cortex, as well as a decrease or increase (in accordance with the objective of the reappraisal technique) of activity in brain regions involved in emotion processing, such as the amygdala and insula (for reviews on the neural correlates, see Ochsner, 2005; Kalisch, 2009; Etkin et al., 2011; Kanske et al., 2011). Another line of research has demonstrated that linguistic processing of the affective aspects of a stimulus can disrupt negative affect (Hariri et al., 2000; Lieberman et al., 2007, Lieberman, 2011). Affect labeling compared to the labeling of facts while experiencing an emotional event reduces amygdala activity. At the same time, activity in the right ventrolateral prefrontal cortex has been shown to increase through affect labeling. This region is involved in inhibiting emotional experience and is associated with the symbolic processing of emotional information. Lieberman et al. (2007) suggested that through affect labeling, language and other symbolic processes could tap into more basic mechanisms of limbic control (e.g., extinction learning). Affect labeling is thought to enhance exposure-related extinction learning effects and to cause unintentional down-regulation of emotion. Interestingly, Lieberman et al. (2011) also showed that in spite of the above mentioned neural evidence to the contrary, subjects did not expect or believe that affect labeling is useful for the down-regulation of negative affect.

Emotion verbalization, that is, verbally confirming that one is feeling something, is usually embedded in a social context. Experiencing emotion promotes social interaction by spurring people’s need to *verbally express and communicate their feelings* to each other. According to Rimé (2009), sharing emotion and receiving social responses, such as empathy and sympathy, serve important hedonic and functional goals, such as stimulating the cognitive processing of a given situation, strengthening interpersonal relationships, and social integration, as well as producing collective meaning and social knowledge. Nils and Rimé (2012) also showed that during emotion sharing, emotional experience varied in accordance with a listener’s response mode. Subsequent to watching an emotion-eliciting movie, subjects sharing their emotion with a listener offering a socio-affective response as opposed to a neutral listener reported higher emotional arousal and more negative valence. Hence, in apparent contradiction with Lieberman et al. (2007), Nils and Rimé found that socio-affective sharing did not alleviate the emotional response, which was only effectively down-regulated by a cognitive sharing mode that included a reframing of the upsetting stimulus. However, after the experiment, participants rated socio-affective sharing as helpful, even though their valence and arousal ratings during the experiment indicated the opposite. Considering these two important studies, it appears that the effects of emotion verbalization are possibly quite complex and not fully understood yet. Assuming that both studies produced valid results, the question arises how these seemingly contradictory results can be integrated. Potential starting points may arise from variations in methodology and parameters, time points of measurements, as well as the employment of different emotion regulation strategies (i.e., affect labeling versus socio-affective sharing). Different verbalization strategies may well have entirely different emotional consequences. Nonetheless, speaking about emotion can evidently modulate emotional experience, reflected in both emotional valence and arousal. In addition, these results indicate that emotion and emotion regulation research benefit from employing social and inter- as well as intra-individual perspectives.

Emotion verbalization is a response-focused emotion regulation strategy. This strategy combines certain aspects of some of the emotion regulation strategies referred to in the first paragraph. It alters the focus of attention and involves cognitive and linguistic processes that help to reappraise the situation. At the same time, it effects emotion expression or suppression. In analogy to the “facial feedback hypothesis,” stating that facial movements can influence emotional experiences (for a classical review, see Fehr and Stern, 1970), it can be assumed that different strategies of verbalization also have an impact on emotional experience. Although verbalizing may be related to various emotion regulation strategies, it does not seem to be equivalent to any one particular strategy, such as reappraisal, suppression, affect labeling, or attention deployment. There are close parallels to affect labeling as described by Lieberman et al. (2007), insofar that both strategies require recognizing one’s own emotional state and verbally attesting to it. However, affect labeling requires identifying the exact emotion (e.g., anger or sadness), whereas verbalizing only demands a general awareness that one is experiencing an emotion. On the other hand, when including denial of the emotional experience as an additional component, verbalizing also involves an aspect of attesting to one’s own emotional state truthfully or misleadingly. Purposefully denying one’s emotion in a conversation may bring about different emotional consequences than generally talking about one’s own emotion. Specifically investigating the role of negation on emotion processing, Herbert et al. (2011) found that negating unpleasant nouns (such as “no fear” compared to “my fear”) decreased emotional arousal ratings and inhibited the startle reflex of the eye. The startle reflex has been associated with emotion processing, insofar as it is generally attenuated by the processing of pleasant stimuli and enhanced by unpleasant stimuli (Lang et al., 1990). Thus, the results by Herbert et al. (2011) indicate that negating an unpleasant noun diminishes the emotional response to that noun. This also fosters the assumption that denying one’s emotion might reduce arousal and modulate emotion, as there are parallels between negating emotional nouns and denying an emotional experience.

To complement and expand on the various study results summarized above, our study aimed at investigating how different verbalization strategies influence emotion, reflected in subjective experience, voice parameters, and skin conductance. To our knowledge, there are no studies scrutinizing the effects of verbal emotion regulation strategies combining the physiological measures of voice and skin conductance response (SCR) during emotion processing. Introducing these physiological parameters in addition to self-report might help to clarify apparent contradictions in previous study results. Specifically, we were interested in exploring the different effects of speaking about emotion versus facts as well as of admitting or denying currently experienced emotion.

To investigate these questions, we showed participants emotion inducing pictures and asked them to verbally admit or deny an emotional experience or a neutral fact concerning the picture. We used a 2 × 2 factorial design manipulating the focus (on emotion or facts) as well as the congruency (admitting or denying) of the verbal expression. We simulated a social emotion-sharing situation through presenting participants with recorded questions pertaining to their emotion, which they answered according to experimental instructions under the different conditions. During the different verbalization conditions, we measured SCR as one indicator of emotional arousal. SCR has been used in a number of studies focusing on emotion regulation. Studies on reappraisal, for instance, report that emotional down-regulation is accompanied by a decrease in SCR (e.g., Egloff et al., 2006; Driscoll et al., 2009; Urry et al., 2009). Previous studies have also shown that the concurrent presentation of affective words during exposure to aversive pictures can diminish SCR (Tabibnia et al., 2008).

Since changes in emotional state are generally accompanied by changes in the laryngeal tension and subglottal pressure in the vocal production system (Schirmer and Kotz, 2006), we also analyzed three parameters of the voice (pitch, voice quality, and average volume) during the different verbalization strategies. Human beings are able to produce highly differentiated sounds (by altering volume, pitch, and spectral energy of different frequency bands, etc.) to communicate more information than the bare words which are being said (Banse and Scherer, 1996). Correspondingly, it is possible for humans to distinguish between different emotions of an interlocutor just by the sound of the voice (Luo and Fu, 2007). These facts evidently demonstrate a link between voice parameters and emotion, which is further backed up by the finding that it is possible to measure the emotional state of a person with regard to valence and arousal by analyzing his or her voice (Scherer, 2003). In an emotionally aroused state, the pitch of the voice is higher (Laukka et al., 2005; Goudbeek and Scherer, 2010). Furthermore, voice volume increases in connection with negative emotional valence (Schröder et al., 2001; Laukka et al., 2005). Scherer (1986) reported that spectral distribution of energy varies significantly with manipulations of intrinsic pleasantness. In line with Scherer (1986), Johnstone et al. (2005) found that listening to unpleasant sounds led to less energy in low frequencies in the voice. These findings suggest that a verbal strategy effectively regulating arousal and/or valence is accompanied by changes in pitch as well as other changes in voice quality and volume.

Based on the literature mentioned above (Hariri et al., 2000; Lieberman et al., 2007), we assumed that talking about emotion in contrast to talking about facts (factor focus) would reduce autonomic arousal, indicated by a physiological response (lower pitch and lower SCR). In addition, we expected congruency (admitting or denying) to exert an effect on autonomic arousal, depending on whether facts or emotion were admitted or denied (interaction between focus factor and congruency factor). Specifically, denying facts was expected to result in a heightened autonomic response (higher pitch and higher SCR) compared to admitting facts, based on study results showing a larger SCR when participants concealed information (Gamer et al., 2007, 2008). In contrast, denying emotion was expected to result in weaker autonomic arousal (lower pitch and lower SCR) than admitting emotion. The latter hypothesis was based on the above mentioned findings by Herbert et al. (2011), and on the assumption that, at least in the present experimental setting, the effect of focus on emotion versus facts would outweigh the effect of congruency, since “lying,” that is, denying facts, was encouraged by the experimental procedure. Thus, the down-regulating effect of verbalizing emotion should be stronger than the up-regulating effect of denial. Other parameters of the voice, such as intensity and voice quality, were to be examined on an exploratory basis. We further assumed that subjects would not consider talking about emotion as being a useful strategy for the down-regulation of emotion based on the findings by Lieberman et al. (2011). A summary of our hypotheses can be seen in Table 1.

**Table 1:** **Expected results (effects of emotional picture and emotion regulation strategies) on pitch and SCR**.

**EFFECTS OF EMOTIONAL PICTURE**	
Picture	Assumed effect of factor picture
Emotional pictures versus neutral pictures	Higher arousal (pitch/SCR) Lower arousal (pitch/SCR)

**EFFECTS OF EMOTION REGULATION STRATEGIES**	
**Focus**	**Assumed effect of factor focus**

Focus on emotion versus focus on facts	Lower arousal (pitch/SCR) Higher arousal (pitch/SCR)

**Congruency**	**Assumed effect of factor congruency**

Admitting emotion versus denying emotion	Higher arousal (pitch/SCR) Lower arousal (pitch/SCR)
Admitting facts versus denying facts	Lower arousal (pitch/SCR) Higher arousal (pitch/SCR)

## Materials and Methods

### Participants

Thirty subjects participated in the study (age range: 21–35 years, *M* = 26.2, SD = 2.98). Half of them were female. Due to technical problems during data recording, post-ratings for one participant, voice data for one participant, and skin conductance data for four subjects were lost. In addition, three participants had to be excluded from SCR data analysis because they lacked a distinct SCR. In sum, *N* = 29 subjects were available for self-report data analyses and voice data analyses and *N* = 23 subjects for skin conductance data analyses.

The study was approved by a local ethics committee and conducted in accordance with the Declaration of Helsinki. Subjects were paid for their participation and gave written informed consent prior to investigation.

### Procedure

Participants were shown into a quiet room and seated comfortably in front of a computer screen with a distance of 0.6 m. Prior to the experiment, participants completed a practice session with similar stimulus material, but only including neutral pictures, to become familiar with the task. The main experiment, which then followed, took about 60 min and consisted of two sessions with a break in between. In total, the experiment contained 108 trials (18 trials per condition), which were presented in a randomized order. During the experiment, we measured the influence of the different verbalizing strategies on parameters of the voice (pitch, intensity, voice quality) and SCR as dependent variables.

To measure the effectiveness of the verbalization strategies with regard to emotion regulation, immediately after the experiment participants were asked to rate on a 9-point scale how much their emotional arousal increased or decreased in each condition (admitting or denying emotion or facts). We cannot rule out that asking subjects for an overall efficiency rating of each strategy after the experiment, and not on a trial-by-trial basis, might limit the informative value of the self-report data. On the other hand, it is assumed that a rating on a trial-by-trial basis might trigger other evaluation processes, such as secondary self-reflection and recollection of feelings during the actual regulation, and might therefore induce confounding effects on arousal (Erk et al., 2010).

### Task

To investigate the impact of different verbalization strategies on emotion processing and regulation, we presented participants with pictures inducing negative emotion and instructed them to respond in the following ways: in the *congruent emotion verbalization condition* (1 – Emo con, emotion admitting), participants were asked to verbally confirm experiencing an emotional reaction elicited by the negative emotional picture (“Correct, I do feel something looking at this picture!” see Table 2 for examples). In the *incongruent emotion verbalization condition* (2 – Emo incon, emotion denying), participants had to verbally deny any emotional response to a picture known to elicit negative emotion (“No, I do not feel anything looking at this picture!”). In the *congruent fact verbalization conditions* (3 – Facts con, 5 – Neut pic con), participants were asked whether or not they see somebody in the picture. They were instructed to answer truthfully and according to what was depicted: “Correct, I do see someone in this picture!” or: “Correct, I do not see anyone in this picture!” In the *incongruent fact verbalization conditions* (4 – Facts incon, 6 – Neut pic incon), participants were instructed to answer incorrectly (i.e., to claim the opposite): “No, I do see someone in this picture!” (even though the image did not show anyone) or: “No, I do not see anyone in this picture!” (even though the image did show a person). Conditions were presented in short blocks of three trials each. Each block was preceded by an instruction cue for 2 s, which stated “emotion admitting,” “emotion denying,” “fact admitting,” or “fact denying,” respectively. For an overview about tasks and conditions, see Table 2.

**Table 2:** **Overview about design and stimulus material**.

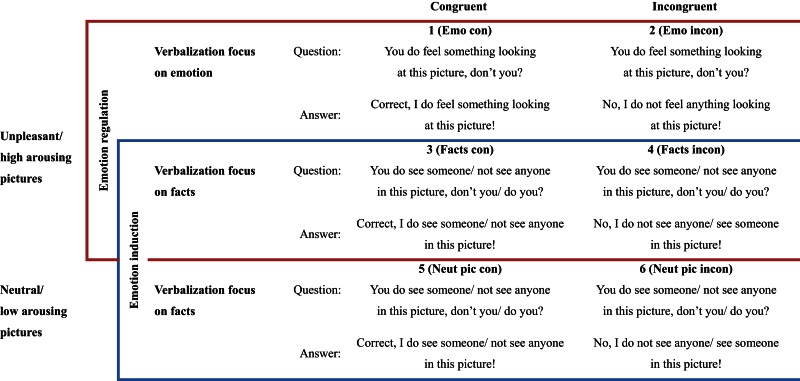

*To investigate effects of emotion regulation within our experimental paradigm, we employed a 2 × 2 factorial design. The conditions used in the emotion induction model are indicated by a blue frame, conditions used in emotion regulation are indicated by a red frame. Conditions 1–4 (with unpleasant pictures) were used to investigate the effect of emotion regulation through different strategies of verbalization. Conditions 5–6 were used as control conditions to test whether the presentation of emotional pictures resulted in emotion induction (during the picture presentation phase) in contrast to neutral pictures (blue). Please note that we used German word material in our study*.

To make the task more interactive and structurally closer to a conversation, we presented one of the following questions during the picture display: “You do feel something looking at this picture, don’t you?” or “You do see someone in this picture, don’t you?” These questions were presented both visually (written below the picture) and acoustically (via headphone). A male and a female speaker each read half of the questions. Pictures and sentences were presented using Presentation^®^ running on a Microsoft Windows operating system (Neurobehavioral Systems Inc., Albany, CA, USA). Acoustic presentation was done via a headset, which also recorded verbal responses given by the participants.

**Figure 1:**
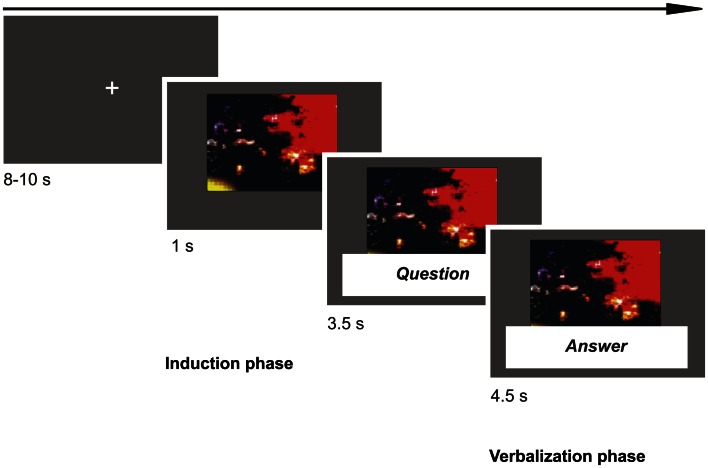
**The figure shows a schematic illustration of an experimental trial**. First, a picture (emotional or neutral) appeared on the screen for 1 s (*induction phase*). Then, the question asking to verbalize in four different ways following our different conditions was presented for 3.5 s (in written form below the picture and verbally via headphone). Then, the answer sentence in red ink appeared below the picture and the participant was given 4.5 s to speak the answer out loud (*verbalization phase*). At the end of a trial, we presented a fixation cross for variable duration (8–10 s) to allow the skin conductance response to recover.

Each trial started with a picture appearing on the screen (*emotion induction phase*). After 1 s of picture presentation, the question was presented for 3.5 s (in written form below the picture and verbally via headphone). Then, the predefined answer sentence appeared below the picture in red ink and the participant was given 4.5 s to speak the answer out loud (*verbalization phase*). Each trial ended with a fixation cross for 8–10 s (*M* = 9 s) to allow the SCR to recover (Dawson et al., 2000; *jittered* *relaxation phase*). Participants were instructed to reply instantly and aloud and as convincingly as possible, and not to look at the answer sentence below the picture too often. Participants were told that the purpose of the study was to investigate emotion processing, and they were aware that their verbal responses were recorded for later analysis. See Figure 1 for a schematic illustration of an experimental trial.

### Stimuli

Pictures were taken from the International Affective Picture System (IAPS; Lang et al., 2005) based on their mean normative ratings for valence and arousal given in the technical manual. We selected 72 unpleasant and emotionally arousing pictures (valence: *M* = 2.74, SD = 0.96; arousal: *M* = 6.05, SD = 0.69) and 36 neutral pictures (valence: *M* = 5.29, SD = 0.59; arousal: *M* = 3.05, SD = 0.56), with a total range of 5.52 for valence and a total range of 5.54 for arousal on a scale from 1 (very unpleasant and no arousal) to 9 (very pleasant and high arousal). Mean valence and arousal ratings of emotional and neutral pictures differed significantly [valence: *t*(106) = −14.49, *p* < 0.001; arousal: *t*(106) = 22.74, *p* < 0.001]. Negative pictures displayed threatening or disgusting scenes, i.e., wild animals, snakes, spiders, corpses, wounded or emotionally distressed people, natural disasters, and accidents. Neutral pictures showed household objects, harmless animals, people at work, social gatherings, buildings, landscape, or portraits.

Pictures were divided into six sets (i.e., into one set for each condition: four emotional and two neutral sets). Each set comprised 18 pictures and was randomly assigned to one of the conditions for each participant. Half of the pictures in each set depicted people or had a social content, half of them did not. Valence did not differ within the two neutral and the four negative sets [negative: *F*(3, 68) = 0.31, *p* = 0.82; neutral: *F*(1, 34) = 0.57, *p* = 0.46]. This also applied to arousal [negative: *F*(3, 68) = 0.13, *p* = 0.94; neutral: *F*(1, 34) = 0.16, *p* = 0.69], and picture luminance [negative: *F*(3, 68) = 0.12, *p* = 0.95; neutral: *F*(1, 34) = 0.06, *p* = 0.81]. Luminance was derived mathematically from the composite color signal of each picture.

All sentences utilized in the experiment (questions and answers) were syntactically identical and had the same number of syllables (see Table 2).

#### Skin conductance recording and analysis

We recorded SCR continuously during the experiment with a sampling frequency of 40 Hz using a commercial skin conductance sampling device (Biofeedback 2000^X-pert^, Schuhfried GmbH, Austria). Skin conductance data were processed using Matlab 7.1 (The MathWorks, Inc., MA, USA). For each trial, we calculated the area under curve separately for the emotion induction and the regulation phases (see Figure 1). Time frame of analysis was 4.5 s, starting from the onset of the picture or answer phase. Each phase was baseline corrected using a period of 200 ms before either the picture or answer onset.

#### Audio recording and analysis

To achieve the best possible results concerning the audio data, we isolated the computer used for stimulus presentation by wrapping the table under which it stood in silence cloth. Furthermore, we used a highly directional headset microphone (AKG C520L Headset, Harman International Industries, Inc., CT, USA), which ensured the voice recordings remained clean as possible by canceling out most of the background noise. The microphone was connected to a handheld recorder (Zoom H2, Zoom Co., Tokyo, Japan) with its output connected to the stimulus presentation computer. For each trial, recording of the voice started 4.5 s after the picture appeared on the screen.

To prepare the recorded voice material for analysis, we first cut out all parts of the spoken sentences that were not identical within the six experimental conditions, leading to short audio clips only containing the end of the sentence (i.e., only the words: “this picture”). Then, we used seewave (Sueur et al., 2008), a package for R Statistics, to compute the following three measurements: pitch (fundamental frequency), intensity, and voice quality. The voice quality was assessed analyzing the frequency spectrum. The spectral analysis returned 256 single frequencies, which were then collapsed, resulting in 11 frequency bands (cf. Banse and Scherer, 1996): 80–125 Hz (s125), 125–200 Hz (s200), 200–300 Hz (s300), 300–500 Hz (s500), 500–600 Hz (s600), 600–800 Hz (s800), 800–1000 Hz (s1000), 1000–1600 Hz (s1600), 1600–5000 Hz (s5000), 5000–8000 Hz (s8000), and 8000–23000Hz (s23000).

### Statistical analyses

All analyses of physiological and rating data were conducted using the R 3.1 statistical package (R Development Core Team, 2012). The ratings were standardized on a within-subject basis; that is, each subject’s responses were converted to standard scores (*M* = 0, SD = 1). This procedure eliminates between-subjects variability, so that subsequent analyses reflect only within-subject variation.

All vocal parameters were normally distributed. SCR data, in contrast, showed a positively skewed distribution and were log transformed. General linear mixed effects models (ANOVA, with subject as a random effect) were calculated on mean level of SCR and the voice parameters: pitch, intensity, and voice quality (intensity of the 11 frequency bands; Pinheiro and Bates, 2002).

For each parameter we used two different random intercept models to analyze our physiological data. Both models consisted of two levels: the upper level representing the subject, and the lower level representing single trial data. The first model tested the effect of emotion induction. It contained the factor picture type (emotional or neutral picture), and included only data from the fact verbalization conditions (see Table 2): 3 (Fact con), 4 (Fact incon), 5 (Neut pic con), and 6 (Neut pic incon). To measure the effect of emotion induction, we compared SCR and voice data of conditions 3 + 4 (Fact con + Fact incon) versus 5 + 6 (Neut pic con + Neut pic incon), isolating the factor picture type. For this model, SCR data was taken from the moment of picture presentation (*emotion induction phase*), before participants started speaking. Evidently, voice data could only be taken from the *verbalization phase*. The second model was used to test for an effect of the different strategies on emotion, and contained the factors focus (facts or emotion) and congruency (admitting or denying). This second model included only conditions during which participants were presented with emotional pictures: 1 (Emo con), 2 (Emo incon), 3 (Fact con), and 4 (Fact incon).

## Results

### Self-report data

**Figure 2:**
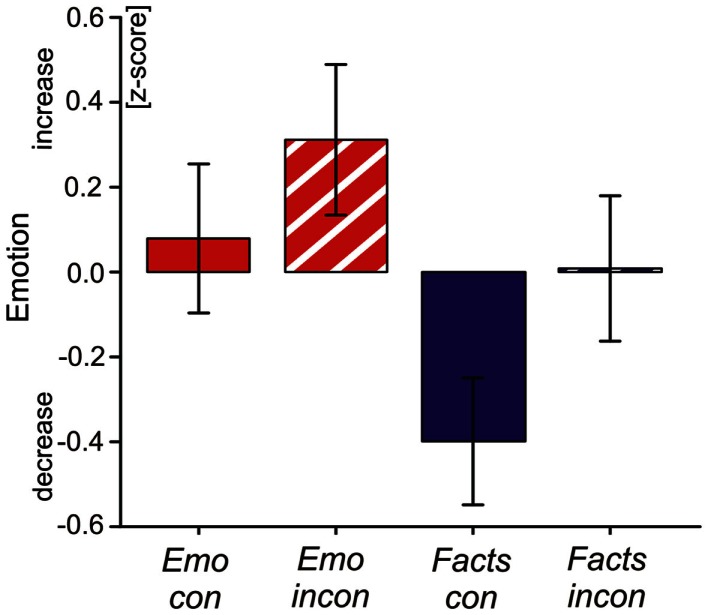
**The figure shows standardized subjective ratings (*z*-scores) of emotion regulation efficacy by verbalization strategies: emotion admitting (Emo con), emotion denial (Emo inc), fact admitting (Facts con), fact denial (Facts inc)**. After the experiment, participants were asked how much each condition subjectively increased or decreased emotional arousal elicited by the pictures on a scale from −4 to +4.

After the experiment, participants reported that talking about facts was, in their opinion, more effective at regulating their emotion than talking about their emotional experience [main effect factor focus: *F*(1, 83) = 5.19, *p* < 0.05]. There was a marginal effect of congruency [*F*(1, 83) = 3.40, *p* = 0.06], indicating that participants perceived congruent verbalization conditions as more effective in down-regulating their emotion than incongruent verbalization conditions (see Figure 2). We found no interaction between focus and congruency [*F*(1, 81) = 0.25, *p* = 0.61].

### Effects of emotional pictures on skin conductance and voice

We first compared SCR (log transformed parameter area under the curve in μS × s) of neutral and negative pictures in the emotion induction phase (0–4.5 s) under the fact verbalization condition. The random intercept model with the factor picture type revealed higher SCR for negative pictures [*F*(1, 1632) = 18.282, *p* < 0.001; Emo pic: *M* = 0.71, SE = 0.005; Neut pic: *M* = 0.68, SE = 0.004] (see Tables 3 and 4).

**Table 3:** **Results from the random-intercept models of the voice analysis for emotion induction (effect of picture) and regulation (effect of strategy)**.

*M* (SE)	Induction		Regulation			
	Neut pic	Emo pic	Emo con	Emo incon	Fact con	Fact incon
SCR	*0.68 (0.004)*	*0.71 (0.005)*	*0.71 (0.01)*	*0.709 (0.007)*	*0.718 (0.007)*	*0.722 (0.007)*
Pitch	*230.407 (1.263)*	*230.271 (0.33)*	*229.439 (0.489)*	*228.81 (0.69)*	*230.379 (1.261)*	*230.156 (0.488)*
s125	52.538 (0.798)	52.401 (0.104)	52.733 (0.143)	53.192 (0.202)	52.412 (0.834)	52.393 (0.142)
s200	58.297 (0.504)	58.349 (0.085)	59.313 (0.124)	59.707 (0.175)	58.528 (0.545)	58.177 (0.124)
s300	57.042 (0.344)	57.219 (0.109)	58.912 (0.161)	59.173 (0.228)	57.518 (0.375)	56.929 (0.161)
s500	51.33 (0.446)	51.613 (0.115)	53.354 (0.166)	53.339 (0.234)	51.882 (0.478)	51.351 (0.166)
s600	46.915 (0.536)	47.209 (0.114)	48.661 (0.168)	48.671 (0.238)	47.409 (0.569)	47.013 (0.168)
s800	37.03 (0.474)	37.258 (0.109)	38.083 (0.161)	38.388 (0.227)	37.415 (0.502)	37.104 (0.161)
s1000	29.928 (0.376)	30.074 (0.123)	30.524 (0.183)	30.965 (0.258)	30.178 (0.397)	29.972 (0.183)
s1600	24.234 (0.393)	24.366 (0.12)	24.597 (0.182)	24.985 (0.257)	24.391 (0.427)	24.342 (0.182)
s5000	20.895 (0.475)	21.005 (0.099)	20.962 (0.149)	21.018 (0.211)	21.065 (0.494)	20.946 (0.149)
s8000	12.683 (0.542)	12.691 (0.1)	12.217 (0.154)	12.677 (0.217)	12.717 (0.55)	12.665 (0.153)
s23000	2.644 (0.992)	2.587 (0.096)	2.245 (0.142)	3.066 (0.201)	2.603 (1.005)	2.571 (0.142)
Intensity	*47.678 (0.135)*	*47.741 (0.047)*	*48.063 (0.066)*	*48.38 (0.093)*	*47,814 (0.151)*	*47,669 (0.066)*

*This table shows mean and standard error (reported in parentheses). A model for each vocal parameter: 80–125 Hz (s125), 125–200 Hz (s200), 200–300 Hz (s300), 300–500 Hz (s500), 500–600 Hz (s600), 600–800 Hz (s800), 800–1000 Hz (s1000), 1000–1600 Hz (s1600), 1600–5000 Hz (s5000), 5000–8000 Hz (s8000), 8000–23000Hz (s23000). SCR [μS] during induction (0–4.5 s), SCR [μS] during regulation (4.5–9.5 s), pitch, and intensity as main parameters are in highlighted in italics*.

**Table 4:** **Results from the random-intercept models of the voice analysis (*F*-values)**.

	Induction	Regulation		
	Factor picture	Factor focus	Factor congruency	Factors focus × congruency
SCR	*F*(1, 1632) = 18.282, *p* < 0.001	*F*(1, 1630) = 4.84, *p* < 0.05	*F*(1, 1630) = 0.095, *p* = 0.75	*F*(1, 1630) = 0.397, *p* = 0.528
Pitch	*F*(1, 2058) = 0.172, *p* = 0.679	*F*(1, 2060) = 10.987, *p* < 0.001	*F*(1, 2060) = 1.52, *p* = 0.218	*F*(1, 2060) = 0.347, *p* = 0.556
s125	*F*(1, 2058) = 1.731, *p* = 0.188	*F*(1, 2060) = 30.996, *p* < 0.001	*F*(1, 2060) = 4.774, *p* < 0.05	*F*(1, 2060) = 5.62, *p* < 0.05
s200	*F*(1, 2058) = 0.373, *p* = 0.542	*F*(1, 2060) = 174.56, *p* < 0.001	*F*(1, 2060) = 0.059, *p* = 0.808	*F*(1, 2060) = 18.018, *p* < 0.001
s300	*F*(1, 2058) = 2.623, *p* = 0.105	*F*(1, 2060) = 255.631, *p* < 0.001	*F*(1, 2060) = 2.075, *p* = 0.15	*F*(1, 2060) = 13.948, *p* < 0.001
s500	*F*(1, 2058) = 6.074, *p* < 0.05	*F*(1, 2060) = 218.147, *p* < 0.001	*F*(1, 2060) = 5.43, *p* < 0.05	*F*(1, 2060) = 4.831, *p* < 0.05
s600	*F*(1, 2058) = 6.634, *p* < 0.01	*F*(1, 2060) = 149.927, *p* < 0.001	*F*(1, 2060) = 2.624, *p* = 0.105	*F*(1, 2060) = 2.919, *p* = 0.088
s800	*F*(1, 2058) = 4.402, *p* < 0.05	*F*(1, 2060) = 74.067, *p* < 0.001	*F*(1, 2060) = 0.001, *p* = 0.98	*F*(1, 2060) = 7.373, *p* < 0.01
s1000	*F*(1, 2058) = 1.399, *p* = 0.237	*F*(1, 2060) = 26.942, *p* < 0.001	*F*(1, 2060) = 0.824, *p* = 0.364	*F*(1, 2060) = 6.268, *p* < 0.05
s1600	*F*(1, 2058) = 1.215, *p* = 0.271	*F*(1, 2060) = 10.974, *p* < 0.001	*F*(1, 2060) = 1.75, *p* = 0.186	*F*(1, 2060) = 2.887, *p* = 0.089
s5000	*F*(1, 2058) = 1.222, *p* = 0.269	*F*(1, 2060) = 0.02, *p* = 0.887	*F*(1, 2060) = 0.089, *p* = 0.765	*F*(1, 2060) = 0.696, *p* = 0.404
s8000	*F*(1, 2058) = 0.006, *p* = 0.938	*F*(1, 2060) = 5.019, *p* < 0.05	*F*(1, 2060) = 3.543, *p* = 0.06	*F*(1, 2060) = 5.594, *p* < 0.05
s23000	*F*(1, 2058) = 0.359, *p* = 0.549	*F*(1, 2060) = 0.489, *p* = 0.484	*F*(1, 2060) = 15.412, *p* < 0.001	*F*(1, 2060) = 18.006, *p* < 0.001
Intensity	*F*(1, 2058) = 1.816, *p* = 0.178	*F*(1, 2060) = 57.889, *p* < 0.001	*F*(1, 2060) = 0.68, *p* = 0.41	*F*(1, 2060) = 5.171, *p* < 0.05

*Effects of picture and strategy*.

In the vocal measures, a significant difference between neutral and emotional pictures appeared in the lower frequency bands [s500: *F*(1, 2058) = 6.074, *p* < 0.05; s600: *F*(1, 2058) = 6.634, *p* < 0.01; s800: *F*(1, 2058) = 4.402, *p* < 0.05]. We found more energy in the lower bands of the frequency spectrum during presentation of negative pictures. We found no differences in intensity [*F*(1, 2058) = 1.82, *p* = 0.178] and pitch with regard to picture type [*F*(1, 2058) = 0.17, *p* = 0.679] (see Tables 3 and 4).

### Effect of verbalization on skin conductance and voice

The random intercept model for SCR in the emotion regulation phase (4.5–9.5 s) included the factors focus and congruency and revealed a main effect of focus in SCR data [*F*(1, 1630) = 4.84, *p* < 0.05], but neither an effect of congruency [*F*(1, 1630) = 0.095, *p* = 0.75] nor an interaction between focus and congruency [*F*(1, 1630) = 0.397, *p* = 0.528]. That is, participants showed lower SCR when verbalizing their emotional experience compared to verbalizing facts (see Table 3).

To test the different effects of verbalization strategies on voice data, we used random intercept models comparing the four conditions that contained emotional pictures (emotion regulation). The models showed main effects of focus in pitch, intensity, and voice quality. During emotion verbalization, pitch was lower [*F*(1, 2060) = 10.987, *p* < 0.001] and voice intensity was higher [*F*(1, 2060) = 57.889, *p* < 0.001] compared to fact verbalization. Additionally, there were effects for the following voice quality parameters: s125, s200, s300, s500, s600, s800, s1000, s1600, s8000. That is, energy in these frequency bands was higher during emotion verbalization (Emo con + Emo incon) compared to fact verbalization (Fact con + Fact incon). Furthermore, the analysis of voice data revealed an effect of congruency in voice quality. The distribution of the frequency spectrum displayed less energy in the very low and very high frequencies (s125, s23000) during congruent verbalizations (Emo con + Fact con), compared to incongruent verbalizations (Emo incon + Fact incon), while energy in the frequency band s500 was increased. There was also an interaction between focus and congruency in intensity in a range of frequency bands (s125, s200, s300, s500, s800, s1000, s8000, s23000; see Tables 3 and 4). The effects were significant for factor focus in the congruent and in the incongruent condition. Tukey’s *post hoc* comparison showed that there was significantly more energy in the s200, s300, and s500 frequencies when participants admitted facts versus denying them, while there was less energy in the very low frequency bands s125 and s200 and the very high frequency bands s8000 and s23000 when participants admitted emotion versus denying them. There was no interaction between focus and congruency in pitch.

## Discussion

In the present study, we investigated how different strategies of verbalization influence emotion processing and regulation in an experimental setting similar to a conversation. We were able to identify effects of the different strategies on SCR, characteristics of the voice, and on self-report data regarding the subjective effectiveness of the different verbalization strategies.

### Emotion induction: Effects of emotional pictures

The presentation of negative as compared to neutral pictures led to an increase in SCR preceding the verbalization phase. As a large number of studies have shown that emotional arousal elicited by affective pictures can be measured in the electrodermal response (Fowles, 1980; Lang et al., 1993), it can be concluded that emotion induction through visual stimulus material was successful in our study.

We also found an effect of negative emotional pictures on voice parameters. As described earlier, the distribution of energy in the frequency spectrum reflects voice quality or timbre (Banse and Scherer, 1996). Participants’ voices displayed a difference in the lower frequency bands (s500, s600, s800) while verbalizing facts, depending on whether they were shown negative or neutral pictures (see Table 3). However, in contrast to other studies (Scherer, 1986; Johnstone et al., 2005) in which less energy in low frequency bands has been associated with unpleasantness of the stimuli, we found more energy in the lower frequencies in response to negative pictures (s500, s600, s800). We cannot rule out that this dissenting finding might be due to language differences (German versus French), controlled speech use versus free speech use, or the analysis of only the last few words of a sentence versus the whole sentence.

### Emotion regulation: Effects of verbalization

Arousal level of participants as indicated by SCR data was modulated by the type of verbalization (see Table 3). SCR was lower when the focus of verbalization was on emotion (i.e., when participants admitted or denied an emotional response to the picture) compared to facts. The same effect of emotion verbalization was also visible in voice parameters. Analysis of voice data showed that pitch was attenuated during emotion verbalization, indicating lower arousal compared to the conditions in which subjects focused on facts (Ladd et al., 1985). The lower skin conductance and lower pitch (see Table 3) during the verbalization of emotion correspond to the results by Tabibnia et al. (2008), suggesting regulatory effects of emotion verbalization similar to the affect labeling mechanism described by Lieberman et al. (2007).

These results are also in line with the findings by Mendolia and Kleck (1993) who compared talking about emotion with talking about the sequence of events of a movie presented previously. The authors showed that subjects who talked about their emotion after watching the movie showed lower autonomic arousal when they viewed the movie a second time 48 h later. In the same study, a reverse effect was found when the second presentation of the movie occurred shortly after the intervention (talking about emotion or facts), indicating that the effect of verbalization is not constant over time. In the present study, we investigated changes in physiological responses at the very moment of the verbalization and found that autonomic arousal was lower for emotion verbalization than for fact verbalization. Hence, the combination of the present results and the findings by Mendolia and Kleck (1993) indicates a U-shaped time course of the regulatory effect of emotion verbalization, insofar that this effect seems to be initiated immediately upon verbalization onset, reverses shortly afterward for a yet unclear period of time, and finally recuperates. This is an interesting thought, as this time course may reflect cognitive or emotional processing induced by the verbalization of emotion. It may be worthwhile to further explore the exact temporal dynamics of this effect of emotion verbalization in future studies.

The effects of emotion verbalization on the physiological indicators of arousal also correspond to the findings by Lieberman et al. (2007), who reported diminished amygdala activity during affect labeling. While utilizing slightly different verbal strategies, our study taken together with the studies by Lieberman et al. (2007) and Mendolia and Kleck (1993) provide cumulative evidence that verbalization of the emotional experience can exert a regulating effect on emotion. Greenberg (2004) argued that putting emotion into words allows an experience to be assimilated into people’s conscious conceptual understanding of the self and the world, and therefore might be a necessary tool of emotion focused therapy. On the other hand, the results provided by Nils and Rimé (2012) suggest that emotion verbalization is not always helpful for resolving negative emotion. Again, differences in time point of measurement and parameters may be responsible for these different findings. Alternatively, it is possible that talking about emotion is beneficial compared to not talking about emotion (and speaking about something else instead). Talking about emotion with an interlocutor giving socio-affective support, however, is less effective for emotional recovery than with an interlocutor encouraging cognitive reframing.

An interesting point is that, in conflict with the physiological evidence, participants reported increased emotional arousal in the emotion verbalization conditions as compared to the fact verbalization conditions (see Table 3). Thus, even though SCR data clearly showed that participants’ autonomic arousal was reduced by emotion verbalization, they seemed to neither notice nor believe that verbalizing their emotion could have this effect. Lieberman et al. (2011) observed the same contradictory results investigating the effects of affect labeling: although affect labeling led to lower distress during the experiment, participants did not believe that affect labeling is an effective emotion regulation strategy. Similarly, Nils and Rimé (2012) also observed that self-report ratings during the experiment stood in direct contradiction with self-report ratings at the end of the experimental procedures with regard to effectiveness of an emotion regulation strategy. These repeated findings invite doubtful speculation on the accuracy of self-reports pertaining to emotional arousal. It is conceivable that participants based their judgment on preconceptions regarding what kind of strategies are generally considered helpful or not helpful when dealing with emotion, rather than introspection. Alternatively, another explanation might arise from different effects of verbalization strategies on emotional valence and arousal. Specifically, we found lower voice intensity during fact verbalization compared to emotion verbalization (see Table 3). Since Scherer (2003) connected an increase of voice intensity to negative valence, we interpret this finding as evidence that fact verbalization led to less negative valence as compared to emotion verbalization. Voice quality measures indicated the same effect. We found less energy in the lower frequencies of the voice for the fact verbalization conditions. Emotion verbalization thus seems to have diminished arousal more than fact verbalization, whereas fact verbalization seems to have reduced negative valence. Subjects might have perceived the changes in valence during fact verbalization as being more important for emotional down-regulation than the changes in arousal during emotion verbalization. Thus, they may have rated fact verbalization as more effective for this reason.

Our results show that emotional responses can be influenced by verbalization, and that emotion is reflected in prosody. Studies comparing the beneficial effects of writing and talking about emotion (Donnelly and Murray, 1991; Harrist et al., 2007) found that participants’ mood was more negative after expressive writing than after talking. The authors concluded that above the effect of expressing one’s emotion in general, vocal expression has an impact on emotion processing. Izard (1990) states that expressive behavior might amplify as well as down-regulate emotional responses. According to Leventhal’s model (Ahles et al., 1983; Leventhal, 1984; Leventhal and Scherer, 1987), verbal expressions stimulate or suppress imagery and expressive motor responses that can alter the emotion associated with the event in question. Leventhal added a feedback loop from automatic facial expressive activity to his perceptual motor model of emotion, which postulates that motor activity modulates emotional experience. A study by Davis et al. (2010) provided evidence for a facial feedback loop by showing that reduced facial expressions after the injection of BOTOX^®^ diminished subjective emotional experience. Adapting Leventhal’s perceptual motor model of emotion, we assume that differences in prosody not only indicate emotional states, but are also perceived by the individual speaking and interact via an auditory feedback loop with emotional experience. We therefore think that prosody of the verbal expression might be an additional factor influencing emotion processing. Prosodic components of speech might contribute to the process of emotion regulation in addition to semantic cognitive components. The idea of an auditory feedback loop is also used in verbal self-monitoring models (e.g., Fu et al., 2006), according to which verbalization transiently activates the speaker’s auditory cortex very early, around 100 ms, after voice onset (Curio et al., 2000). The verbal denial of emotion might also have initiated response tendencies similar to those induced by the suppression of emotional expression. Expression suppression is an emotion regulation strategy described by Gross (1998a,b, 2007). Verbal expressions presumably include not only facial but also lingual motor responses. This might lead to a mixed physiological state including increased sympathetic activation due to the additional task of suppressing behavioral response tendencies (Gross, 2007).

We also found a main effect for the factor congruency in the distribution of energy in the frequency spectrum (see Tables 3 and 4; s125, S500, s23000). We found more energy in the very low (s125) and very high (s23000) frequency bands during the denial conditions, while there was less energy in the s500 frequency bands. Since we had no specific hypotheses regarding the distribution of energy in the frequency spectrum for congruency, these findings have to be considered exploratory. We did hypothesize an interaction between focus and congruency in pitch and SCR, since lying about facts has been associated with an increase in physiological arousal (Gamer et al., 2007, 2008) while denying emotions has been associated with a decrease (Herbert et al., 2011). We did not find an interaction in pitch and SCR but in voice intensity and voice quality in both high and low frequency bands (s125, s200, s300, s500, k800, k1000, k8000, k23000). In line with Hirschberg et al. (2005), these results suggest that voice parameters might be useful for detecting deception. At the same time, our data indicates that denying an emotional experience exerts different physiological consequences than denying facts. Possibly, when it comes to talking about emotion, this effect of denying might be reversed by a potentially stronger effect of emotion verbalization. However, that idea needs to be further explored by future studies.

### Limitations

Since every language has specific characteristics, we cannot rule out that our findings might be influenced by characteristics of the German language, or culture specific language use. Considering that the voice changes over lifespan, our results only refer to young speakers (aged from 20 to 30 years), and cannot be generalized to other age periods.

We cannot rule out that constraining the efficacy rating of each strategy to one point in time after the experiment, as opposed to a trial-by-trial rating, might have limited the informative value of the self-report data. On the other hand, it has been argued that ratings on a trial-by-trial basis can trigger other evaluation processes, such as secondary self-reflection and recollection of feelings during the actual regulation, and might therefore cause confounding effects on arousal (Erk et al., 2010). Nevertheless, for future research we recommend a trial-by-trial rating, bearing in mind the possible implications.

We think that further research is needed to assess how verbalizing affects the different dimensions of emotion, and to replicate our findings, since parts of the study were exploratory. We also recommend assessing both valence and arousal, since different verbalization strategies seem to have different effects on both dimensions.

In the present study, we investigated the regulatory effect of different verbal strategies on skin conductance and voice parameters by using only negative or neutral pictures. Therefore, our results cannot be generalized to emotional stimuli with positive valence.

We found effects in voice parameters indicating a modified emotional state. Given the paradigm of our study, we were unable to test whether the differences in voice parameters are noticeable to a listener and thus impact social interactions (cf. Johnstone et al., 2005). This question could be addressed in further studies by letting a second group of subjects rate the recorded responses of the first subject group regarding the emotional state of the speaker (valence and arousal).

## Conclusion

Our experiment focused on the sender’s side of communication and investigated through which channels emotion is transmitted, and how emotion is modulated during the act of speaking. We thereby contribute to a more comprehensive understanding of how emotion is communicated, even if the person in question tries to deny his or her emotional state. According to Rimé’s (2009) approach, people feel the need to convey their emotion. One explanation emerging from our results for this need to communicate emotion could be that people verbalize their emotion on purpose in an attempt to regulate it. We found evidence suggesting that the different strategies of verbalization employed in the experiment are capable of regulating someone’s emotional state, and that a verbal feedback loop affects emotional experience in a similar way to the facial feedback loop described in Leventhal’s perceptual motor model of emotion (Leventhal, 1984). We would like to draw attention to the selective influence of different verbalization strategies on valence and arousal and to the potential this distinction might have for future research in this field. Specifically, we found that verbalizing one’s emotion affected arousal, while focusing on facts of an emotional event modulated valence.

## Conflict of Interest Statement

The authors declare that the research was conducted in the absence of any commercial or financial relationships that could be construed as a potential conflict of interest.

## References

[B1] Ahles T. A. Blanchard E. B. Leventhal H. (1983). Cognitive control of pain: attention to the sensory aspects of the cold pressor stimulus. Cognit. Ther. Res. 7, 159–177 10.1007/BF01190070

[B2] Banse R. Scherer K. R. (1996). Acoustic profiles in vocal emotion expression. J. Pers. Soc. Psychol. 70, 614–636 10.1037/0022-3514.70.3.614 8851745

[B3] Curio G. Neuloh G. Numminen J. Jousmäki V. Hari R. (2000). Speaking modifies voice-evoked activity in the human auditory cortex. Hum. Brain Mapp. 9, 183–191 10.1002/(SICI)1097-0193(200004)9:4<183::AID-HBM1>3.0.CO;2-Z 10770228 PMC6871984

[B4] Davis J. Senghas A. Brandt F. (2010). The effects of BOTOX injections on emotional experience. Emotion 10, 433–440 10.1037/a0018428 20515231 PMC2880828

[B5] Dawson M. E. Schell A. M. Filion D. L. (2000). “The electrodermal system,” in Handbook of Psychophysiology, 2nd Edn, eds Cacioppo J. T. Tassinary L. G. Bernston G. G. (Boston: Cambridge University Press), 200–223

[B6] Demaree H. Robinson J. Pu J. (2006). Strategies actually employed during response-focused emotion regulation research: affective and physiological consequences. Cogn. Emot. 20, 1248–1260 10.1080/02699930500260427

[B7] Donnelly D. A. Murray E. J. (1991). Cognitive and emotional changes in written essays and therapy interviews. J. Soc. Clin. Psychol. 10, 334–350 10.1521/jscp.1991.10.3.334

[B8] Driscoll D. Tranel D. Anderson S. W. (2009). The effects of voluntary regulation of positive and negative emotion on psychophysiological responsiveness. Int. J. Psychophysiol. 72, 61 10.1016/j.ijpsycho.2008.03.012 18845192 PMC2676237

[B9] Egloff B. Schmukle S. C. Burns L. R. Schwerdtfeger A. (2006). Spontaneous emotion regulation during evaluated speaking tasks: associations with negative affect, anxiety expression, memory, and physiological responding. Emotion 6, 356–366 10.1037/1528-3542.6.3.356 16938078

[B10] Erk S. Mikschl A. Stier S. Ciaramidaro A. Gapp V. Weber B. (2010). Acute and sustained effects of cognitive emotion regulation in major depression. J. Neurosci. 30, 15726–15734 10.1523/JNEUROSCI.1856-10.2010 21106812 PMC6633759

[B11] Etkin A. Egner T. Kalisch R. (2011). Emotional processing in anterior cingulate and medial prefrontal cortex. Trends Cogn. Sci. 15, 85–93 10.1016/j.tics.2010.11.004 21167765 PMC3035157

[B12] Fehr F. S. Stern J. A. (1970). Peripheral physiological variables and emotion: the James-Lange theory revisited. Psychol. Bull. 74, 411–424 10.1037/h0032958 5496761

[B13] Fowles D. C. (1980). The three arousal model: implications of gray’s two-factor learning theory for heart rate. Psychophysiology 17, 87–104 10.1111/j.1469-8986.1980.tb00117.x 6103567

[B14] Fu C. H. Y. Vythelingum G. N. Brammer M. J. Williams S. C. R. Amaro E. Jr. Andrew C. M. (2006). An fMRI study of verbal self-monitoring: neural correlates of auditory verbal feedback. Cereb. Cortex 16, 969–977 10.1093/cercor/bhj039 16195470

[B15] Gamer M. Bauermann T. Stoeter P. Vossel G. (2007). Covariations among fMRI, skin conductance, and behavioral data during processing of concealed information. Hum. Brain Mapp. 28, 1287–1301 10.1002/hbm.20343 17290371 PMC6871443

[B16] Gamer M. Verschuere B. Crombez G. Vossel G. (2008). Combining physiological measures in the detection of concealed information. Physiol. Behav. 95, 333–340 10.1016/j.physbeh.2008.06.011 18638496

[B17] Goudbeek M. Scherer K. (2010). Beyound arousal: valence and potency/control cues in the vocal expression of emotion. J. Acoust. Soc. Am. 128, 1322–1336 10.1121/1.3466853 20815467

[B18] Greenberg L. S. (2004). Emotion-focused therapy. Clin. Psychol. Psychother. 11, 3–16 10.1002/cpp.387 19639649

[B19] Gross J. J. (1998a). The emerging field of emotion regulation: an integrative review. Rev. Gen. Psychol. 2, 271–299 10.1037/1089-2680.2.3.271

[B20] Gross J. J. (1998b). Antecedent- and response-focused emotion regulation: divergent consequences for experience, expression, and physiology. J. Pers. Soc. Psychol. 47, 224–237 9457784 10.1037//0022-3514.74.1.224

[B21] Gross J. J. (2007). “Emotion regulation: conceptual foundations,” in Handbook of Emotion Regulation, ed. Gross J. J. (New York: Guilford Press), 3–24

[B22] Hariri A. R. Bookheimer S. Y. Mazziotta J. C. (2000). Modulating emotional responses: effects of a neocortical network on the limbic system. Neuroreport 11, 43–48 10.1097/00001756-200001170-00009 10683827

[B23] Harrist S. Carlozzi B. L. McGovern A. R. Harrist A. W. (2007). Benefits of expressive writing and expressive talking about life goals. J. Res. Pers. 41, 923–930 10.1016/j.jrp.2006.09.002

[B24] Herbert C. Deutsch R. Sütterlin S. Kübler A. Pauli P. (2011). Negation as a means for emotion regulation? Startle reflex modulation during processing of negated emotional words. Cogn. Affect. Behav. Neurosci. 11, 199–206 10.3758/s13415-011-0026-1 21369874

[B25] Hirschberg J. Benus S. Brenier J. M. Enos F. Friedman S. Gilman S. (2005). “Distinguishing deceptive from non-deceptive speecho,” in Proceedings of the 9th European Conference on Speech Communication and Technology (Interspeech ’05), Lisbon, 1833–1836

[B26] Izard C. E. (1990). Facial expressions and the regulation of emotion. J. Pers. Soc. Psychol. 58, 487 10.1037/0022-3514.58.3.487 2182826

[B27] Jackson D. C. Malmstadt J. R. Larson C. L. Davidson R. J. (2000). Suppression and Enhancement of Emotional Responses to Unpleasant Pictures. Psychophysiology 37, 515–522 10.1111/1469-8986.3740515 10934910

[B28] Johnstone T. van Reekum C. M. Hird K. Kirsner K. Scherer K. R. (2005). Affective speech elicited with a computer game. Emotion 5, 513 10.1037/1528-3542.5.4.513 16366756

[B29] Kalisch R. (2009). The functional neuroanatomy of reappraisal: time matters. Neurosci. Biobehav. Rev. 33, 1215–1226 10.1016/j.neubiorev.2009.06.003 19539645

[B30] Kanske P. Heissler J. Schönfelder S. Bongers A. Wessa M. (2011). How to regulate emotion? Neural networks for reappraisal and distraction. Cereb. Cortex 21, 1379–1388 10.1093/cercor/bhq216 21041200

[B31] Ladd D. R. Silverman K. E. A. Tolkmitt F. Bergmann G. Scherer K. R. (1985). Evidence for the independent function of intonation contour type, voice quality, and F0 range in signaling speaker affect. J. Acoust. Soc. Am. 78, 435–444 10.1121/1.392466

[B32] Lang P. J. Bradley M. M. Cuthbert B. N. (1990). Emotion, attention, and the startle reflex. Psychol. Rev. 97, 377 10.1037/0033-295X.97.3.377 2200076

[B33] Lang P. J. Bradley M. M. Cuthbert B. N. (2005). International Affective Picture System (IAPS): Affective Ratings of Pictures and Instruction Manual Technical Report A-6. Gainesville, FL: University of Florida

[B34] Lang P. J. Greenwald M. K. Bradley M. M. Hamm A. O. (1993). Looking at pictures: affective, facial, visceral, and behavioral reactions. Psychophysiology 30, 261–273 10.1111/j.1469-8986.1993.tb03352.x 8497555

[B35] Laukka P. Juslin P. Bresin R. (2005). A dimensional approach to vocal expression of emotion. Cogn. Emot. 19, 633–653 10.1080/02699930441000445

[B36] Lazarus R. S. Folkman S. (1984). Stress, Appraisal, and Coping. New York: Springer Publishing Company

[B37] Leventhal H. (1984). A perceptual-motor theory of emotion. Adv. Exp. Soc. Psychol. 17, 117–182 10.1016/S0065-2601(08)60119-7

[B38] Leventhal H. Scherer K. (1987). The relationship of emotion to cognition: a functional approach to a semantic controversy. Cogn. Emot. 1, 3–28 10.1080/02699938708408361

[B39] Lieberman M. (2011). “Why symbolic processing of affect can disrupt negative affect: social cognitive and affective neuroscience investigations,” in Social Neuroscience: Toward Understanding the Underpinnings of the Social Mind, eds Todorov A. Fiske S. Prentice D. (New York: Oxford University Press), 188–209

[B40] Lieberman M. D. Eisenberger N. I. Crockett M. J. Tom S. M. Pfeifer J. H. Way B. M. (2007). Putting feelings into words. Psychol. Sci. 18, 421–428 10.1111/j.1467-9280.2007.01916.x 17576282

[B41] Lieberman M. D. Inagaki T. K. Tabibnia G. Crockett M. J. (2011). Subjective responses to emotional stimuli during labeling, reappraisal, and distraction. Emotion 11, 468–480 10.1037/a0023503 21534661 PMC3444304

[B42] Luo X. Fu Q. (2007). Vocal emotion recognition by normal-hearing listeners and cochlear implant users. Trends Amplif. 11, 301–315 10.1177/1084713807305301 18003871 PMC4111530

[B43] Mendolia M. Kleck R. E. (1993). Effects of talking about a stressful event on arousal: does what we talk about make a difference? J. Pers. Soc. Psychol. 64, 283–292 10.1037/0022-3514.64.2.283 8433274

[B44] Nils F. Rimé B. (2012). Beyond the myth of venting: social sharing modes determine the benefits of emotional disclosure. Eur. J. Soc. Psychol. 42, 672–681 10.1002/ejsp.1880

[B45] Ochsner K. (2005). The cognitive control of emotion. Trends Cogn. Sci. (Regul. Ed.) 9, 242–249 10.1016/j.tics.2005.06.004 15866151

[B46] Pinheiro J. C. Bates D. M. (2002). Mixed-Effects Models in S and S-PLUS. (New York: Springer).

[B47] R Development Core Team. (2012). R: A Language and Environment for Statistical Computing. Vienna: R Foundation for Statistical Computing

[B48] Rimé B. (2009). Emotion elicits the social sharing of emotion: theory and empirical review. Emot. Rev. 1, 60–85 10.1177/1754073908097189

[B49] Scherer K. R. (1986). Vocal affect expression: a review and a model for future research. Psychol. Bull. 99, 143 10.1037/0033-2909.99.2.143 3515381

[B50] Scherer K. R. (2003). Vocal communication of emotion: a review of research paradigms. Speech Commun. 40, 227–256 10.1016/S0167-6393(02)00084-5

[B51] Schirmer A. Kotz S. A. (2006). Beyond the right hemisphere: brain mechanisms mediating vocal emotional processing. Trends Cogn. Sci. (Regul. Ed.) 10, 24–30 10.1016/j.tics.2005.11.009 16321562

[B52] Schröder M. Cowie R. Douglas-Cowie E. Westerdijk M. Gielen S. (2001). “Acoustic correlates of emotion dimensions in view of speech synthesis,” in Proceedings of Eurospeech, Aalborg, Vol. 1. 87–90

[B53] Sueur J. Aubin T. Simonis C. (2008). Seewave: a free modular tool for sound analysis and synthesis. Bioacoustics 18, 213–226 10.1080/09524622.2008.9753600

[B54] Tabibnia G. Lieberman M. D. Craske M. G. (2008). The lasting effect of words on feelings: words may facilitate exposure effects to threatening images. Emotion 8, 307–317 10.1037/1528-3542.8.4.551 18540747 PMC4727455

[B55] Urry H. L. van Reekum C. M. Johnstone T. Davidson R. J. (2009). Individual differences in some (but not all) medial prefrontal regions reflect cognitive demand while regulating unpleasant emotion. Neuroimage 47, 852 10.1016/j.neuroimage.2009.05.069 19486944 PMC2766667

